# The Tongue as a Breeding-Place for Bacteria

**Published:** 1901-12

**Authors:** M. H. Fletcher

**Affiliations:** Cincinnati, Ohio


### THE TONGUE AS A BREEDING-PLACE FOR BACTERIA.[1]

BY M. H. FLETCHER, D.D.S., M.D., M.S., CINCINNATI, OHIO.

It is evident that the surface of the tongue may hold pabulum and protection for the development and growth of myriads of bacteria. It is possible that cavities in the teeth, and gums diseased from deposits about the teeth, are more prolific, but probably not.

Out of the "seven nutrient media for bacteria in the oral cavity" given by Miller, five are always present on a coated tongue, —viz., normal saliva, buccal mucus, dead epithelium, particles of food, and exudations from diseased gums.

Formerly physicians were very careful about the examination of the tongue, but at present the diagnostic value of its coating is largely disputed. On the other hand, some of the best physicians make the tongue the principal basis of their diagnosis, especially in gastro-intestinal troubles. But the tongue as a breeding-place for bacteria is properly attracting more attention at present and cannot justly be ignored.

Van Valzah-Nesbet has said, "The tongue is not a mirror of the stomach, as the ancients supposed, but is a local mirror which reflects only that which comes over its surface."

Dr. A. L. Russell says, "The color is due to the pearly color of the cornified epithelial cells covering the papillæ; to retained particles of food; and the *débris* of micro-organisms, broken-down epithelium, and mucus. The color of the organ proper is quite as important as its motions, contour, lesions, and coating. It is well to remember that in perfect health there is usually a slight, uniform coating of a whitish color diffused over the entire tongue, and that towards the base of the tongue in the region of the circumvallate papillæ there is always a heavy coating which is increased with any increase of the normal coating of the anterior portions."

Hare says, "The coating may be black from the ingestion of iron, charcoal, bismuth, ink-berries, cherries, or red wine. It may be stained brown from tobacco, licorice, nuts, prunes, or chocolate. It may be yellow from the ingestion of rhubarb or laudanum."

---

[1] Abstract of a paper read before the American Medical Association, Section on Stomatology, St. Paul, Minn., June 4, 5, 6, and 7, 1901.

F. Forschheimer says, "In some cases the tongue is of great clinical importance, and in many others its examination for diagnostic purposes is without value. It goes without saying that no case has been completely examined unless the mouth has been looked into, and yet, in a great many cases nothing is gained by this examination. . . . The tongue is affected as the result of local and general conditions. . . . The changes that take place are in the direction of size, shape, color, coating, or fur. The blood affects the color of the tongue, as a whole, more than any other cause.

"The furring of the tongue is that portion of our subject which has been most studied. The fur upon the tongue is, when examined microscopically, seen to be made up of epithelial cells, molecular detritus, and organisms of various kinds, held together by mucus. The organisms are those usually found in the mouth; sometimes we find pathogenic organisms, most frequently the pneumococcus and pus producers."

"In long-continued fevers in which the absence of moisture is the predominating cause we have a peculiar condition of a dry white fur, quite thick and adherent."

"When there is not sufficient movement of the tongue there results a fur, because fewer of the old cells are removed than would be under normal circumstances. In paralytics we constantly see a furred tongue."

"The place of deposit of this fur depends very much upon the size and shape of the tongue; where the tongue does not come in contact with other parts of the mouth it will be thick; at the edge it will be rubbed off, leaving a red outline."

"The most ludicrous mistakes occur to those who overlook the fact that particles of food and medicinal agents give their color to the fur; rhubarb produces a beautiful liver tongue."

The consensus of opinion of the foregoing investigators seems to be that the coatings of the tongue are local and not indicative of any particular disease, and that the mass contains large quantities of bacteria.

These coatings may be accounted for as follows:

The dorsum of the tongue, with its processes, glands, fissures, and rough horny surface, is most favorably constructed for holding detritus from its own surface and from other parts of the mouth, also small particles of food and tartar, but the great factor of the formation and persistence of this coating is mucus. The coating forms at times into almost a membrane. This is due to the cementing qualities and the insolubility of the mucus discharged into the mouth.

The easily and quickly forming cakes of mucus in the nostrils are accounted for by the continuous passage of air over its surface, drying it rapidly, carrying dust and foreign matter into its substance, where it becomes embedded, causing it to be black, yellow, gray, or other color, dependent upon the character of foreign particles with which the air is freighted, the surface exposed to the air becoming hardest; that next to the membrane is softer. When the mucus finally dries and shrinks it breaks into patches, curls, and drops away, or is removed.

The mucus of the mouth when separated from the more fluid portions of the saliva is just as strong a cementing agent as that of the nostrils, and holds the particles of food, dead epithelial cells, and tartar into a mass or layer, covering the rough surface of the tongue, also cementing the coating to the slanting, filiform processes, and to the uneven surface. Under this layer the mucus is continually secreted, and the desquamation of epithelium goes on; thus the layer is continuously added to from below as well as from above.

The upper surface of the layer being constantly wet has no opportunity to become dry and break up into patches as in the nose. In persistent mouth-breathing it may occur, or portions of the coating may be removed mechanically, producing what is called "patchy tongue."

Such systemic disorders as tend to produce fever have their effect upon the mucous membrane, among the first of which is the "dry stage." In this stage the mucus is thicker, less abundant, and dries quicker. This is as true of the dorsum of the tongue as of any other mucous membrane, hence the coating is usually pronounced and well formed by the time the doctor is called to see the patient.

The color of the coating is largely effected by the incorporation of blood from different parts of the mouth, most frequently from bleeding gums, a condition found in a large percentage of the people at all times, or it may be colored from many sources as cited, and not unfrequently from bacteria.

In addition to personal observation the foregoing would seem strong evidence to convince one that the coatings of the tongue are from local causes, but may be favored and in[illegible]sed by febrile conditions which precede or accompany al[illegible]y bodily ailment. Lack of cleanliness and mouth-breathi[illegible]doubted and prolific causes.

The danger from unclean tongu[illegible]hs containing decayed teeth and diseased gums are [illegible]t forth by Miller. He says, "From a neglected mouth, su[illegible]peatedly comes under the observation of dentists, enormous [illegible]tities of bacteria must reach the intestinal tract, in spite of t[illegible]rilization of food. In a very unclean mouth examined for this purpose I estimated by culture methods the number of cultivable bacteria at 1,140,000,000; many of them were doubtless carried to the stomach during every meal, to be replaced by others developed between meals and overnight.

"Von Kaczorowiski proves clearly enough that the micro-organisms in an unclean mouth, quite independent of those introduced with the food and drink, suffice to produce intense fermentative processes, chronic dyspepsia, etc."

Even with decayed teeth and diseased gums to breed bacteria in great numbers, the dorsum of the tongue still presents the largest surface for their growth; and if it is not cleaned once or twice a day (which practically no one does) it is one of the greatest of all places in the mouth for breeding bacteria.

The mucus which cements this layer of sordes upon the tongue is not soluble in water, ether, alcohol, chloroform, or dilute mineral acids, but is soluble in alkaline solutions, and since nearly all of our food is either acid or neutral, and not one in thousands thinks of scrubbing the tongue as a matter of cleanliness and protection, it certainly is a source of danger and infection, and an undoubted condition for perpetuating disease, especially with invalids.

In order to test the solubility of mucus in alkaline solutions, try scrubbing the tongue with a tooth-brush filled with powdered biborate or bicarbonate of soda, and notice how the coat disappears. (Biborate is the least disagreeable.) Protrude the tongue and scrub it from back to front until the coating is removed. When the tongue is to be examined for diagnostic purposes this should be done so that the real color and condition of the tongue can be examined. Then the diagnostic features will be visible and of undoubted value. The use of the brush is much better than to scrape the tongue with a hard instrument. Ordinary tooth-powders are not satisfactory, because the finely pulverized chalk or other earthy matter remains on the tongue like food and dead epithelium, and the real condition and color of its surface is still unexposed for inspection. Coarse powdered borax or cornmeal, or the two mixed, form a coarse detergent powder very efficient for this purpose, as well as for cleaning the teeth and gums.

A clean mouth undoubtedly prevents much fermentative and putrefactive indigestion, not to mention its comfort and the prevention of tooth-decay and diseased gums.

Animals keep deposits from the tongue by its use as a prehensile organ, and by licking their bodies and other substances. Then the saliva of animals in their native state is decidedly alkaline in reaction, and keeps the mucus dissolved much better than does the human saliva. The latter is either acid or very weak in alkaline reaction, a condition accompanying civilized life, and may be found in animals kept as pets or in confinement and fed upon prepared foods.

There is probably no way of estimating the great immunity brought about by keeping the mouth and all it contains perfectly clean. As a prophylactic measure no physician can afford to neglect it, either for himself or his patients.

